# Gene expression profiling of protease and non-protease genes in *Trichophyton mentagrophytes* isolates from dermatophytosis patients by qRT-PCR analysis

**DOI:** 10.1038/s41598-020-79839-1

**Published:** 2021-01-11

**Authors:** Shyama Datt, Shukla Das, M. Ahmad Ansari, Rahul Sharma, Thakur Datt, S. N. Bhattacharya

**Affiliations:** 1 Department of Microbiology, University College of Medical Sciences and Guru Teg Bahadur Hospital, Delhi, 110095 India; 2 Department of Community Medicine, University College of Medical Sciences and Guru Teg Bahadur Hospital, Delhi, 110095 India; 3 Department of Dermatology, University College of Medical Sciences and Guru Teg Bahadur Hospital, Delhi, 110095 India; 4 Molecular Oncology Laboratory, Delhi State Cancer Institute Dilshad Garden, Delhi, 110095 India

**Keywords:** Applied microbiology, Fungi, Microbiology, Pathogenesis

## Abstract

*Trichophyton mentagrophytes* secretes Metallocarboxypeptidase A and B of the M14 family as endoproteases and exoprotease. *T. mentagrophytes* produce Metalloprotease 3 and 4 which degrades the protein into the short peptides and amino acids. To understand the host fungal relationship and identification of such genes expressed during infection is utmost important. *T. mentagrophytes* encodes some proteins which are associated with the glyoxylate cycle. The glyoxylate cycle enzymes have been involving in virulence of dermatophytes and their up-regulation during dermatophytes growth on keratin. On comparing the expression level of virulence protease and non-protease genes, we observed, among exoprotease protease genes, Metallocarboxypeptidase B was strongly up regulated (134.6 fold high) followed by Metallocarboxypeptidase A (115.6 fold high) and Di-peptidyl-peptidases V (10.1 fold high), in dermatophytic patients as compared to ATCC strain. Furthermore, among endoprotease, Metalloprotease 4 was strongly up regulated (131.6 fold high) followed by Metalloprotease 3 (16.7 fold high), in clinical strains as compared to *T. mentagrophytes* ATCC strain. While among non-protease genes, Citrate Synthase was highly expressed (118 fold high), followed by Isocitrate Lyase (101.6 fold high) and Malate Synthase (52.9 fold high). All the studied virulence genes were considered the best suitable ones by geNorm, Best keeper, Norm Finder and Ref finder.

## Introduction

Dermatophytosis is a common superficial fungal infection of skin, hair and nail. Though not a part of the normal human microbial flora, the fungus is well adapted to infecting the host keratin^1^. Chronic dermatophytic infection has emerged as a major public health problem amongst the superficial fungal infections. For the survival of the fungus in the presence of antifungal, various compensatory mechanism exist which allow the fungus to adapt to the hostile environment during the course of infection^2^. Currently, recalcitrant dermatophytosis not responding to Terbinafine and other antifungals has increasingly been reported and attributed to emergence of drug target alteration in ergosterol biosynthesis^3^.

For establishment of infection *T. mentagrophytes* demonstrates adherence of arthroconidia at 6 h of contact with stratum corneum followed by germination of spores, while on a nail plate model, the adherence and germination of *T. mentagrophytes* arthroconidia were seen after 6 h post-inoculation and the growing hyphae were identified 10 h later. Adherence of *T. mentagrophytes* was also investigated using an ex-vivo model made of human skin explants of full epidermis demonstrating maximal adherence at 12 h, with and penetration of the stratum corneum after 3 days^4^. Having established adherence to the surface, dermatophytes secrete keratinase, which decomposes human and animal keratin^3^. In-vitro demonstration of substantial proteolytic activity, in a medium containing protein, utilizing it as the source of nitrogen and carbon, establishes the role of enzymes in initiating infection. The proteases in dermatophytic infection are widely recognized, and these enzymes have been identified as important virulence determinants and allergens^5^. It is estimated that proteases represent approximately 20% of the 100 most expressed secreted proteins in *T. benhamiae* (or *Arthroderma benhamiae*) during growth both in-vitro and in-vivo. Other dermatophytes like *T. verrucosum* including *T. benhamiae* were found to own 235 predicted protease-encoding genes, none unique to either species^6^.

Dermatophytes release endoprotease that cleave peptide bonds within a polypeptide and exoprotease cleave peptide bonds only at the N- or the C-terminae of polypeptides^7,8^. Serine protease, leucine aminopeptidases (LAP1 and LAP2) and metalloprotease (MEP) are the predominant endoproteases, though exoproteases such as aminopeptidase, metallocarboxypeptidase (MCP) and dipeptidyl-peptidases (DPP-IV and DPP-V), have also been isolated from dermatophytes^9^. Overall there are multiples endoproteases that have been broadly categorized into two major protein families, the subtilisins*,* that are serine proteases (SEP), and the fungalysin or metalloprotease (MEP). Amongst the Metalloprotease Giddey et al. (2007) experimentally found high secretion levels of MEP3 and MEP4, in the culture supernatant of *T. mentagrophytes* grown on a soy protein medium, while *T. violaceum* predominantly secreted MEP2 and MEP4^10–13^. Besides these the enzymes involved in glyoxylate cycle are also related to the pathogenicity of *T. mentagrophytes*, though their functions and mechanisms remain uncertain. The Isocitrate lyase (IL) and Malate synthase (MS), as the key enzymes of the glyoxylate cycle were highly induced in macrophages during *S. cerevisiae* infection^14^^.^

We attempted to analyze the expression patterns and dynamics of genes encoding two major families of endoproteases and exoproteases, which comprised of Metalloproteases (MEP3 and MEP4) Metallocarboxypeptidases (MCP-A and MCP-B) and key enzymes of glyoxylate cycle, Isocitrate lyase (IL), Citrate synthase (CS), Malate synthase (MS) and Dipeptidyl-peptidases (DPP-V), in *T. mentagrophytes* isolates from patients of chronic dermatophytosis by real-time (RT) PCR and compared the expression levels with *T. mentagrophytes* ATCC number 28185 strain^15,16^.

Further, to measure the expression stability of the noted differentially expressed genes which are responsible for degrading keratin into proteins and peptides, different online web based software such as geNorm^17^, BestKeeper^18^, NormFinder^19^ and RefFinder^20^, have been applied to determine and validate the regulation of pathogenic virulence genes in *T. mentagrophytes*.

## Result

### Identification of *T. mentagrophytes* isolates by phenotypic and genotypic methods

A total of 40 clinical isolates of dermatophytes were identified routine phenotypic method and then *T. mentagrophytes* isolates were genotypically confirmed by conventional PCR shown in Fig. 1.1–3. Sequencing was performed to confirm the *T. mentagrophytes* isolates. The representative sequences obtained were submitted to gene bank data base with accession numbers; MH644185, MH745112 and, MH778308.

**Figure 1:**
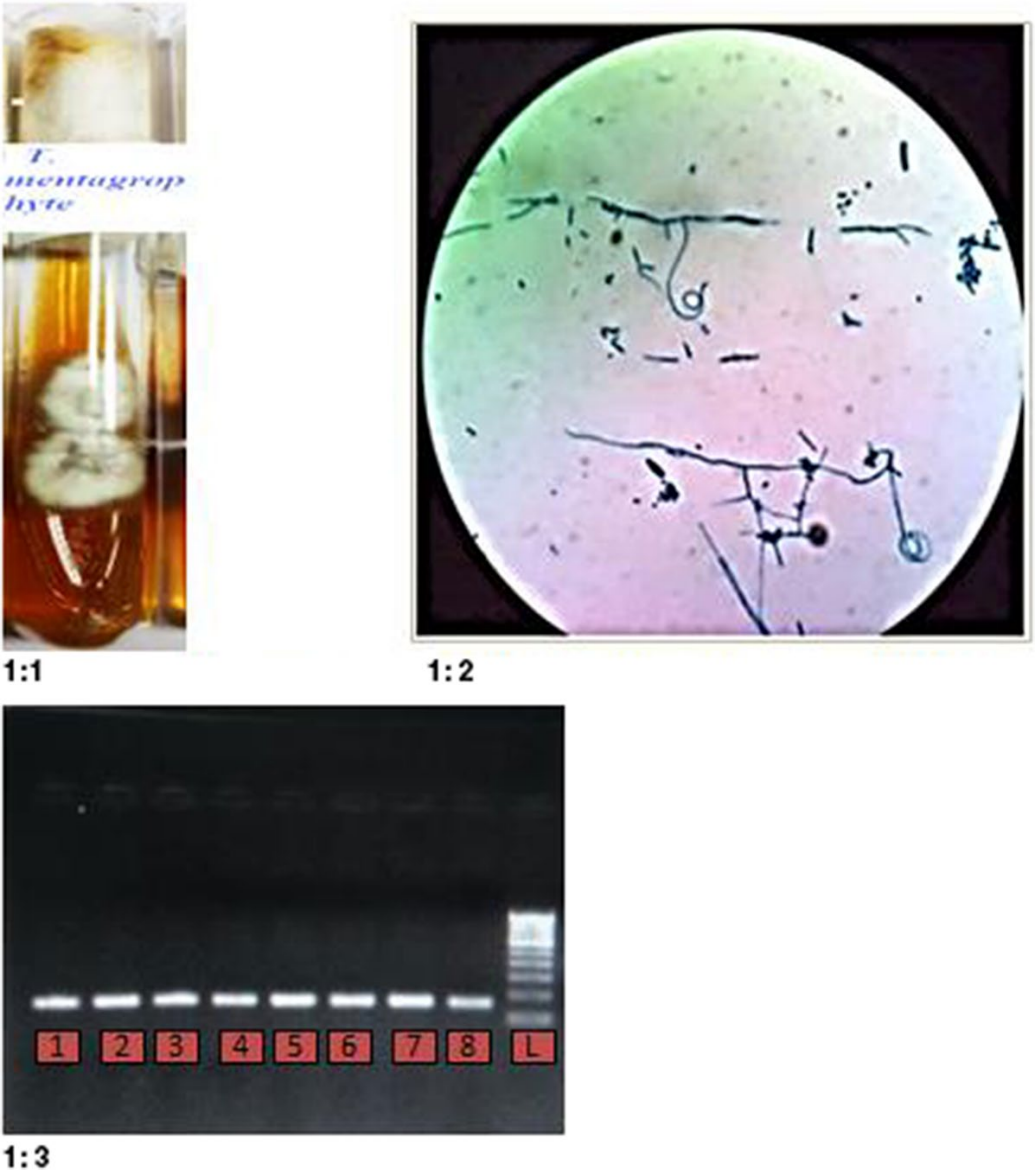
1.1 Culture picture of *T. mentagrophytes*. 1.2 Microscopic picture of *T. mentagrophytes* in LPCB. 1.3 *T. mentagrophytes* picture at 130 base pair Lane 1–8 & L:1–7- samples, 8-ATCC of *T. mentagrophytes* & L-ladder 100 base pair.

The clinical samples included 38 cases of tinea corporis and tinea cruris and 2 of onychomycosis. The mean age and disease duration in these patients was 29.8 ± 11.41 years and 9.74 ± 6.81 months, respectively. Most patients had previously received antifungal therapy but with poor clinical response and currently presented with persistent lesions.

### Expression of virulence genes of *T. mentagrophytes* isolates in dermatophytosis patients

**Figure 2:**
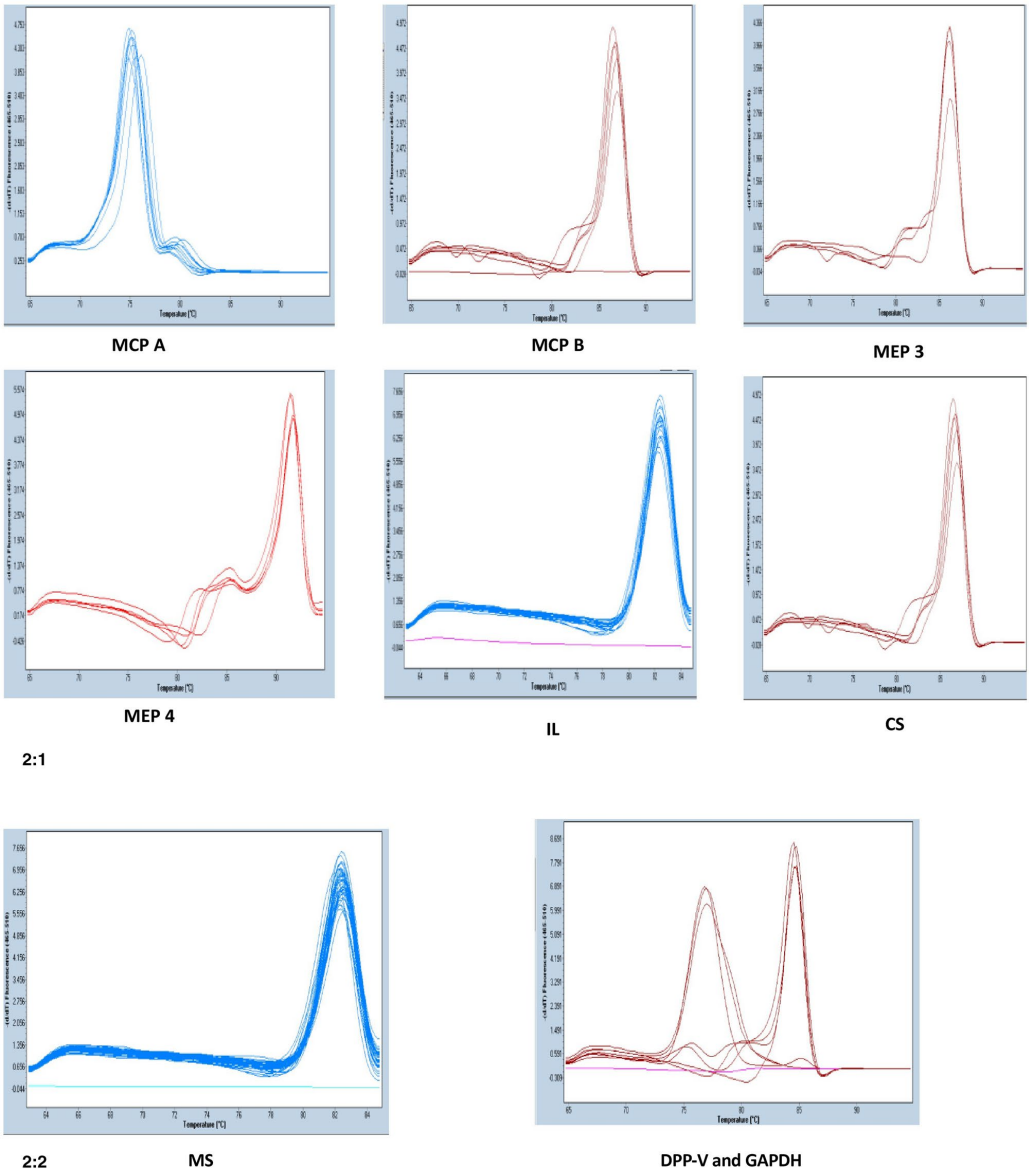
2.1 Melting peak of MCP-A, MCP-B, MEP3, MEP4, IL and CS. 2.2 Melting peak of MS, DPPV and GAPDH.

The real-time PCR analysis of virulence genes has shown that the expression of the most common virulence genes i.e. MEP3, MEP4, MCP-A, MCP-B, IL, CS, MS and DPP-V was up-regulated. The amplification pattern and melting peak of MEP3, MEP4, MCP-A, MCP-B, IL, CS, MS and DPP-V genes are shown in Fig. 2.1,2.

Forty clinical isolates of *T. mentagrophytes* were analysed against ATCC strain and the fold change was calculated. Both the groups were assessed for eight virulence genes were normalized by Glyceraldehyde-3-phosphate Dehydrogenase (GAPDH) shown in Table 1.

**Table 1:** Status of differential expression level of virulence genes of *T. mentagrophytes.*

S. No	Gene	Ct value of clinical isolates (mean ± SD)	Ct value of *T. mentagrophytes* ATCC 28185 (mean ± SD)	Δ Ct value of clinical isolates	Δ Ct value of ATCC	ΔΔ Ct value	Expression level (2^− ΔΔCt^ method)
1	MEP3	27.51 (± 4.90)	34.42 (± 0.24)	− 1.49	− 1.76	0.27	16.7
2	MEP4	28.46 (± 3.84)	30.80 (± 0.29)	− 0.54	1.85	− 2.39	131.6
3	MCPA	29.08 (± 4.03)	34.86 (± 0.22)	0.07	2.30	− 2.22	115.9
4	MCPB	25.34 (± 3.32)	31.61 (± 0.28)	− 3.66	− 0.96	− 2.70	134.6
5	IL	31.33(± 3.34)	35.8(± 0.07)	2.32	3.24	− 0.91	101.6
6	CS	28.48 (± 5.12)	35.94 (± 0.61)	− 0.22	3.38	− 3.60	118.0
7	MS	30.43 (± 4.14)	32.62 (± 0.19)	1.43	0.06	1.37	52.9
8	DPPV	29.66 (± 5.71)	31.69 (± 0.21)	0.66	− 0.88	1.53	10.1

**Figure 3:**
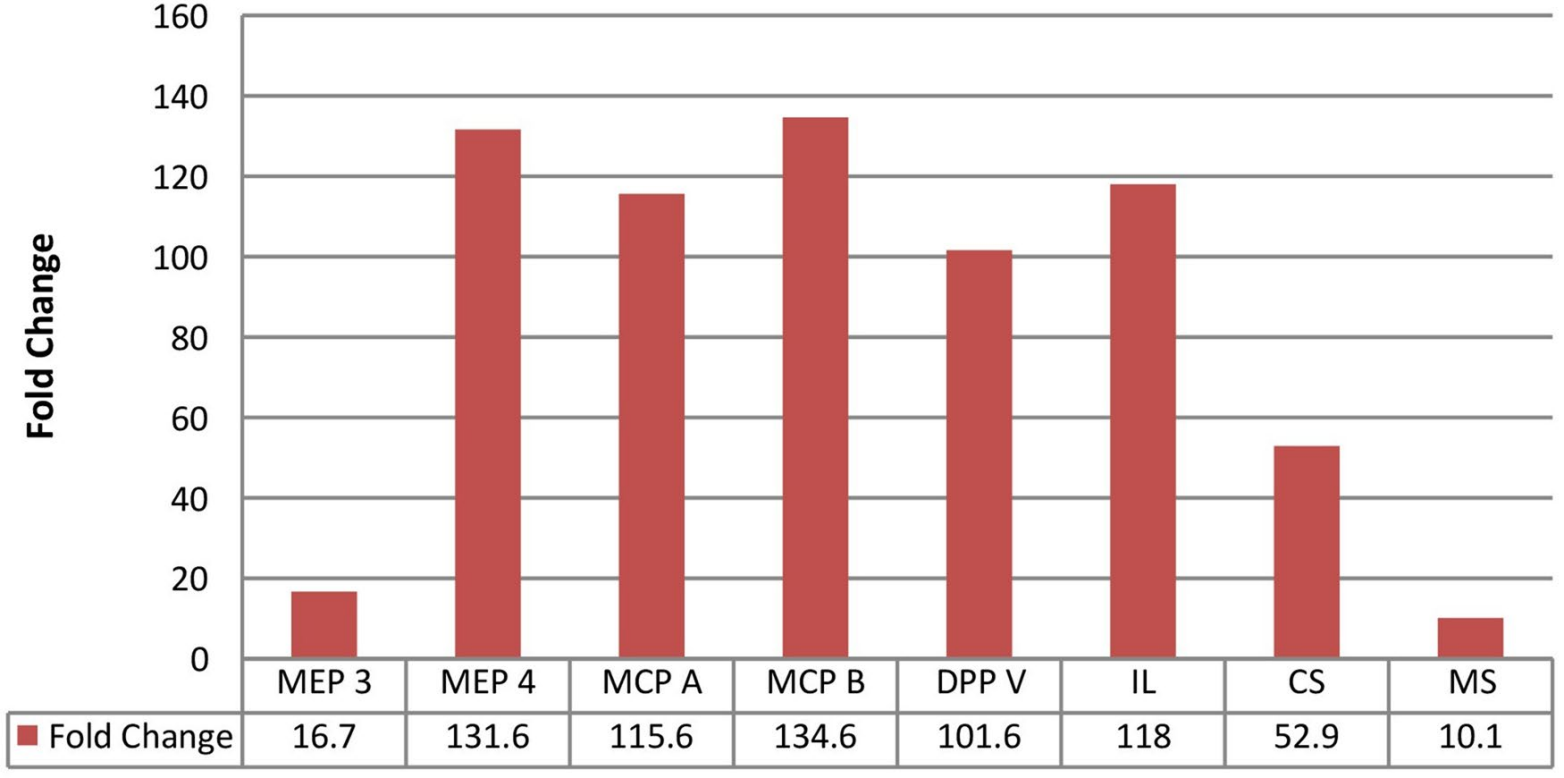
Fold change of all virulence genes.

On comparing the fold change expression level we observed, the major keratinase, MCP-B of the exoprotease family was strongly up-regulated (134.6 fold higher) followed by MCP-A (115.6) and DPP-V (10.1), in *T. mentagrophytes* as compared to ATCC strain. Furthermore, among endoprotease, MEP4 was strongly up regulated (131.6 fold higher) followed by MEP3 (16.7 fold), in clinical isolates. While amongst non-protease genes, Citrate Synthase was highly expressed (118 fold higher), followed by Isocitrate Lyase (101.6) and Malate Synthase (52.9), in *T. mentagrophytes* isolates as shown in Fig. 3.

### Expression stability ranking of virulence gene

The ranking of virulence gene data as per geNorm software depicting the expression stability (M) value from low to high; the NormFinder data with the stability (P) value ranging from low to high; the BestKeeper data demonstrating the coefficient of correlation (r) value from high to low while RefFinder data demonstrating the Geometric mean of ranking values ranging from low to high is shown in Table 2.

**Table 2:** Ranking of Virulence genes of *T. mentagrophytes* on the basis of their expression stability determined by geNorm, NormFinder, BestKeeper, and RefFinder.

Rank	GeNorm		NormFinder		BestKeeper		RefFinder	
	Gene	Expression stability (M)	Gene	Stability value (p)	Gene	Coefficient of correlation (r)	Gene	Geometric mean of ranking values
1	MCP-B	0.273	MCP-B	0.105	DPP-V	0.614	MCP-B	1.32
2	MCP-A	0.294	MCP-A	0.134	MEP4	0.604	MCP-A	2.91
3	MEP4	0.298	MEP4	0.141	CS	0.583	MEP4	3.13
4	IL	0.307	IL	0.148	MCP-B	0.531	IL	4.95
5	CS	0.31	CS	0.153	MCP-A	0.477	MEP3	5.66
6	MEP3	0.33	MEP3	0.176	IL	0.467	CS	5.96
7	MS	0.34	MS	0.185	MS	0.219	MS	7.11
8	DPP-V	0.344	DPP-V	0.189	MEP3	0.121	DPPV	9

The stability analysis with geNorm showed that metallocarboxypeptidases (MCP-A and B) and metalloprotease (MEP4) genes were the more stable genes, with MCP-B ranked as the most stable gene (M value = 0.273) and serine protease DPP-V the least stable gene (M value = 0.344) amongst all virulence genes analyzed in dermatophytosis samples. RefFinder and NormFinder algorithms calculated the expression stability of 8 virulence genes demonstrated MCP-B as the most stable genes and DPP-V as the least stable one. On the contrary, BestKeeper analysis gave the DPP-V as the most stable gene in *T. mentagrophytes* isolates, followed by MEP4 and MEP3 as the least stable gene. Hence, BestKeeper analysis also showed MEP4 with high coefficient of Correlation (r) as most stable gene in *T. mentagrophytes*. Furthermore, this validation analysis using geNorm, NormFinder and RefFinder was confirm that MCP-B, MCP-A and MEP4 genes were expressing consistently and have role in the pathogenesis of *T. mentagrophytes*.

According to geNorm, Norm Finder and Ref Finder algorithm, MCP-B, MCP-A and MEP4 genes were more stable genes followed by IL, while best keeper showed DPP-V as most stable gene along with MEP4, CS and MCP-B amongst the eight genes. Hence, MCP-B, MCP-A and MEP4 genes are the most stable and predictable virulence genes for monitoring host-fungal pathogenicity, as described by geNorm, NormFinder and RefFinder (Table 2).

## Discussion

Dermatophytes are keratinolytic fungi which preferentially survive on keratinocytes. Their anthropophillic adaptation, using a variety of host proteins, particularly keratin, as nutrient source requires secretion of several proteases to degrade skin and hair proteins. The understanding of the pathophysiological mechanisms involved in establishment of dermatophyte infection can give us critical insight for rational management strategies^21^. The level of keratinase secretion by Trichophyton species has been reportedly higher than that of Microsporum species, associating their role in pathogenesis^22^.

To establish infection, the ability of the pathogen to overcome the physical and innate host resistance, and endure the initial struggle to establish infection, while competing with normal microbial flora of skin, are all critical enabling factors. The production of various enzymes subsequently triggers a cascade of reactions, which ultimately favour the survival and growth of the pathogen on the host.

This study has attempted to analyse the change in gene expression, encoding various proteins in *T. mentagrophytes*, causing infection. Certain genes may be overtly engaged in the facilitating the persistence of *T. mentagrophytes* in the host. We observed that the patients harbouring *T. mentagrophytes* for several months, despite antifungal treatment, had persistent lesions. This suggested a possible biological mechanism to evade the host immune system to clear the fungus. The array of protease secreted by dermatophytes for establishing infection provides the necessary environment for the fungus to continue to proliferate and remain infective^23–25^.

According to studies by Zhang et.al and Leng et al. suggested that MEP4 may be the dominant metalloprotease during the host infection stage among the dermatophytes, MEP4 and MEP5 genes have a significant contribution to *T. mentagrophytes* pathogenicity during the host invasion^9,26^. We demonstrated similar findings in our study with high expression of gene coding MEP4, although endoprotease (MCP-A, MCP-B and DPP-V) and non-protease (IL, CS and MS) also demonstrated increased expression, though not to the degree of that of metalloprotease enzymes.

Various researchers have shown that MEP2 and MEP3 genes of *M. canis* were significantly expressed at the time of infection, demonstrated in animal model. Other genes of *M. canis,* such as DPP-IV and DPP-V, may also have specialized functions in the host-fungus relationship^27^. At least 22 distinct genes of *T. mentagrophytes* proteases viz. DPP-V, SUB3, SUB5 MEP3, MEP4, MCP-A and MCP-B etc. are known to be involved in protein digestion^22,27,28^. However, not all keratin-induced proteases play an active role during infection. The zoophilic dermatophyte *Arthroderma benhamiae*, produces inflammatory infection in humans and expresses both endoprotease and exoprotease genes, when grown on keratin-soy medium. The expression of endoprotease genes encoding keratinases, were strongly up-regulated in *A. benhamiae,* with serine proteases (SUB3 and SUB4) and metalloproteases (MEP1, MEP3 and MEP4) as the major classes expressed. Another study by Peter Staib et al. has observed strongly induced MCP-A gene in *A. benhamiae* during infection and growth on keratin^9^, attributing its relevance in pathogenesis. The peptides generated by endoprotease activity are further hydrolysed by exoproteases, followed by uptake of tripeptides, dipeptides and amino acids for use in fungal metabolism. A previous study identified that non-protease genes belonging to Citrate Synthase, Malate Synthase and Isocitrate Lyase were also up-regulated during infection, encoding key enzymes of the glyoxylate cycle by *A. benhamiae*^15^. Little is known about the role of non protease virulence genes of glyoxylate cycle, though their involvement in the adherence process had been demonstrated in *M. canis* and *T. mentagrophytes*^15,29^. In our study we found MEP4 was strongly up-regulated (131.6 fold) as compared to MEP3 (16.7 fold) in *T. mentagrophytes* isolates. Several documented reports have demonstrated a uniform expression of other genes in *T. mentagrophytes* including Dipeptidyl peptidases (DPP-V), Metallocarboxypeptidase (MCP-A and MCP-B), and Serine Carboxypeptidases (SCP B). Secreted proteases are therefore suggested to fulfill other important functions to establish infection that are not exclusively associated with the degradation of keratin^7–9^.

It appears that, variable expression of virulence genes are a result of several factors including growth conditions, and genomic plasticity. During keratin degradation, in dermatophytes several enzymes participate in digestion of host protein. Subtilisins and fungalysins digest proteins into large peptides, which are subsequently broken down into short chain peptides and amino acids. Once cleaved, the reduced proteins become accessible for further digestion by various other endo- and exoproteases secreted by the fungi.

The high expression of MCP-B (134.6 fold) and MCP-A (115.6 fold) by *T. mentagrophytes*, isolated from dermatophytosis patients, was a major highlight of our study. This finding correlates well with studies of Burmester et al.^10^, who found that only some of the typically keratin-induced proteases like MCP-B, secreted from *A. benhamiae*, were strongly expressed during fungus-keratinocyte interaction. We also observed Citrate Synthase was strongly up-regulated (118 fold), along with Isocitrate Lyase (101 fold) and Malate synthase (52.9 fold). The expression of various proteases in clinical isolates of *T. mentagrophytes* play a vital role in survival of the fungus adapting to the new challenging environment, a possible evolutionary mechanism to evade stress and persist on the host skin surface. To validate the stability and expression of various genes the geNorm software, determines the stability value, where in the smaller M-value corresponds to more stable gene. As earlier reported, a comparison on the pathogenic potential of five metalloprotease genes from *T. mentagrophytes* led to the proposal that MEP4 and MEP5 were most likely to affect pathogenicity, determined in a guinea pig model and a keratin degradation test, whereas expression of only MEP4 was significantly up regulated following growth in vitro on keratin, collagen, elastin or human skin sections. When *T. rubrum* was cultured with human keratinocytes (HaCaT), elevated expression of MEP4 and subtilisins genes was observed, whereas in a feline *M. canis* skin infection model there was no evidence of MEP expression during adhesion or early stages of invasion^14,15^.

Our study emphasizes the significance of various proteases though the precise role of these endoproteases, exoprotease and non-protease genes in skin and nail invasion remains to be investigated. Thus *T. mentagrophytes* isolated from patients of dermatophytosis despite antifungal treatment with a failed to clear the fungus indicate a complex phenomenon of survival strategy of a pathogen. The preferential expression of various proteases convey their ability to evade adverse environmental circumstances and overcome the inhibitory effects of antifungals and host defense mechanism, adapt to the hostile environment persist on lesions^1,10^.

Acquisition of drug resistance is a stepwise mechanism and resistance to Terbinafine and high MIC to azoles has been reported in *T. mentagrophyte*. However to achieve better survival, fungi adopt various other mechanism to emerge as dominant species amongst the rest. Hence the role of enzymes may be an important stress adaptation strategies expressed in *T. mentagrophytes.*

## Conclusion

Superficial dermatophytosis is a common fungal infection in humans and exoproteases which are proteolytic enzymes digest hard keratinized structure. This study gives us an insight to the virulence properties of *T. mentagrophytes* responsible for pathogenicity in patients with dermatophytosis. Amidst various factors like rising antifungal resistance, the property of, dermatophytes to secrete several kinds of proteases during penetration, are key factors in the invasion and utilization of peptides on the stratum corneum of the host as a source of energy for their survival. Therefore, an understanding of the specific virulence factors involved in pathogenicity of dermatophytes would assist in the development of new therapeutic approaches to inhibit the proliferation and minimize the inoculum at the site of infection and facilitate the antifungal action on the dermatophytes.

## Materials and methods

Study group: The present study was conducted on 40 patients clinically diagnosed with dermatophytosis, attending dermatology outpatient department of a tertiary care hospital, Delhi during a period, January 2015 to December 2018. The skin and nail samples of these patients were collected, after obtaining written informed consent and obtaining Institutional ethical clearance.

Ethical clearance was obtained from UCMS & GTB Hospital. Helsinki guidelines- the study was performed accordance with the declaration of Helsinki. It is widely regarded as a cornerstone document on human research ethics.

### Screening and identification of isolates

Each clinical specimen was suspended in a drop of 10% potassium hydroxide (KOH) for microscopic examination. A portion of the sample was cultured on Sabouraud’s dextrose agar (SDA, Hi-media; India) with antibiotics Chloramphenicol (0.05 g/l), Gentamicin (20 mg/l) and Cycloheximide (0.5 g/l). All inoculated tubes were incubated at 25 °C for optimal growth up to 4 weeks. In case of growth, the etiological agent was confirmed by the characteristic morphology of the colony, microscopic appearance of the fungus on Lacto Phenol Cotton Blue (LPCB) mount.

### DNA extraction and PCR for molecular confirmation

After 21 days of incubation on SDA, the growth of *T. mentagrophytes* were scrapped off from the agar slants and the cells were suspended in normal saline for further counting in colony counter unit. Approximately, one million cells per ml were taken and centrifuged at 12,000 rpm for 15 min. The pellet was re-suspended in 500 µl of lysis buffer and extraction of DNA was done using standard commercially available kit protocol (Real Biotech Corporation, Taiwan).

The species specific primers^30^ targeting the internal transcribed spacer 2 (ITS 2) of *T. mentagrophytes* were used and was confirmed by a 130 bp PCR product length as shown in Table 3. Each tube contained PCR reaction mixture of 25 μl which included 2.5 μl buffer (10X), 5 μl of Q-buffer, 0.5 μl dNTPs (200 μM), MgCl_2_ 0.5 μl (1.5 mM), 0.15 μl Taq polymerase, 1 μl of each primer, forward and reverse (10 μM), 5 μl of the extracted DNA and nuclease free water to make up the final volume. All PCR reagents were purchased from Qiagen, USA and amplification was performed on a thermo-cycler (Eppendorf, Germany). An initial denaturation was performed at 95 °C for 10 min, followed by 35 amplification cycles of 30 s at 95 °C, 45 s at 65 °C, 30 s at 72 °C, and a final extension of 10 min at 72 °C. The amplified PCR products were analyzed by electrophoresis on 1.5% agrose gel, stained with ethidium bromide and separated at 125 V and 15 mAh current, in a 10 slot apparatus for 30 min. Molecular marker of 100 bp was used to determine the size of the amplicons. Standard ATCC control strain of *T. mentagrophytes* ATCC no. 28185 was used as positive control^31^.

**Table 3:** Details of species specific primer of *T. mentagrophytes.*

Target region	Primer Sequence	Tm	Product size	References
ITS2	Forward 5′-CAA ACG TCC GTC AGG GTG AGC-3′ Reverse 5′-TAG CCA CTA AAG AGA GGC TCG C-3′	65 °C	130 bp	^30^

Purification of PCR products followed by DNA sequence analysis was performed and by comparison of the nucleotides with dermatophytes reference sequences obtained from gene bank data base (http://www.ncbi.nih.gov/gene), the isolates were specified as *T. mentagrophytes* complex which were showing similarity of 99%.

### RNA extraction and complimentary DNA synthesis

Total RNA was extracted from 40 culture isolates of *Trichophyton mentagrophytes* using TRIzol Reagent (Invitrogen, USA). Briefly, 1 ml of TRIzol Reagent was added in the sample and mixed gently by micropipette to form a homogeneous cell lysate and incubated for 5 min at room temperature. 200 µl of chloroform per ml of TRIzol Reagent was added and centrifuged at 12,000 rpm for 15 min at 4 °C to obtain a colorless upper aqueous phase. RNA containing aqueous phase was taken in a fresh 1.5 ml tube and washed with 500 µl of Isopropanol. The eppendorf tube was centrifuged at 12,000 rpm for 10 min at 4 °C and supernatant was discarded. 1 ml of 75% ethanol was added to the pellet and mixed by vortex. This was followed by centrifugation at 10,000 rpm for 5 min at 4 °C. The supernatant was discarded and the pellet was resuspended in RNase free water, and incubated at 55–60 °C for 15 min. The isolated RNA was stored at − 80 °C immediately. The integrity of the RNA samples was confirmed by agarose gel electrophoresis. Concentration and purity of the samples were assessed by Nano-drop (Ependorff, USA). Extracted RNA was used as a template for c-DNA synthesis by Superscript reverse transcriptase II (Invitrogen, USA). Total RNA (2–5 µg) from each sample was reverse transcribed into c-DNA under thermal conditions, 25 °C for 10 min., 37 °C for 120 min., 85 °C for 5 min. and 4 °C for infinite time.

### Expression study of virulence genes by quantitative real-time PCR (qRT-PCR)

To quantify the expression of virulence genes, Metalloprotease 3 (MEP3), Metalloprotease 4 (MEP4), Metallocarboxypeptidase A (MCP-A), Metallocarboxypeptidase B (MCP-B), Isocitrate lyase (IL), Citrate synthase (CS), Malate synthase (MS) and Dipeptidylpeptidase V (DPP-V), qRT-PCR was performed on Light Cycler 480 Instrument (Roche Diagnostics, Germany). Forty confirmed isolates of *T. mentagrophytes* and *T. mentagrophytes* ATCC no. 28185 were screened for virulence gene expression. The final Real-Time PCR reaction of 20 µl contained 2 µl of c-DNA, 10 µl of Syber Green Universal Master Mix, and 60 M of virulence genes primers. The Real Time PCR conditions were standardized according to the melting temperature (Tm) of the primers^8,9^ as shown in Tables 4 and 5. Glyceraldehyde-3-Phosphate Dehydrogenase (GAPDH) served as an endogenous expression control for normalization of data and the threshold cycle (Ct) values were calculated by the software. The dissociation curve analysis was performed to verify that a single product was amplified. Amplification curve and melt peaks for each virulence gene were shown in Fig. 2.1,2. The fold change of the expression of virulence genes of *T. mentagrophytes* in clinical isolates was calculated in compare to ATCC strain by 2^−ΔΔCt^ method^32^.

**Table 4:** Details of virulence genes and their specific primers of *T. mentagrophytes.*

S. no	Gene	Primer sequence	Tm	References
1	**MEP3**	Forward 5′-AGC AGC ACG CCA GCA ACG-3′	60	^8^
		Reverse 5′-GCA GAC GGA AGG ACT CGA TGT-3′		
2	**MEP4**	Forward 5′-AGT CGG GAC ACC ATT CTT CAG-3′	60	^8^
		Reverse 5′-ATT TGG GCT TCT ATG CTC TAC G-3′		
3	**MCPA**	Forward 5′-GCA TTG AAG GCG GTG CAT-3′	60	^9^
		Reverse 5′-GTC AAC ACT GTC TCC ATT AAC TTG GT-3′		
4	**MCPB**	Forward 5′-GTT CTC GAG TGC AGT ATG GCT ACA ACC AG-3′	60	^9^
		Reverse 5′-GTT AGA TCT TAT TTT AAC CTG AAA ATA GGA T-3′		
5	**IL**	Forward 5′-TGG AAA GAT TCA AGA TGG CGA TA-3′	60	^10^
		Reverse 5′-TCT GTG CAT TTG ATG GGT AAT CA-3′		
6	**CS**	Forward 5′-GAA CAT GGT AAA TGG TCA GGT GAA-3′	60	^10^
		Reverse 5′-CGC CGA GGG TGA AGT CAA-3′		
7	**MS**	Forward 5′-TCTTTCTCTCCTCTCTTTCTTTCCTTCCAACCAC-3′	60	^10^
		Reverse 5′-CAG AAG ACG ATT GAC TTG GAA TTT CTC AAG TGC TGT T-3′		
8	**DPP V**	Forward 5′-CTT AGA TCT GTT CCT CCT CGT GAG CCC CG-3′	60	^8^
		Reverse 5′-CTTGCGGCCGCTCATTCCTCTGCCCTCTCACC-3′

**Table 5:** Thermal profile of Metalloprotease 3 and 4 (MEP3 and 4); Metallocarboxypeptidase A and B (MCP A and B); Dipeptidyl-Peptidase V (DPP V); Isocitrate lyase; Citrate synthase; Malate synthase and GAPDH.

Temperature	Time	Cycles	Phase
95 °C	10 min	1	Initial Denaturation
95 °C	20 s	35	Denaturation
**60** °C	**30 s**		**Annealing**
72 °C	20 s		Extension
95 °C	10 s	1	Melting temperature
65 °C	30 s		
95 °C	20 s		
40 °C	30 s		Hold

Bold indicates that at this temperature the PCR is standarized.

### Stability index (SI) analysis

The stability index of virulence genes was calculated using the web based softwares, Best Keeper, geNorm, NormFinder and RefFinder. Best Keeper uses raw data, Ct values and Real time PCR efficiency (r) to determine the expression of pathogenic genes and combines them into an index. The software computes descriptive statistics of all derived Ct values for the eight virulence genes that is the geometric mean, arithmetic mean, minimal and maximal value, standard deviation, and coefficient of variance. The stability of virulence genes expression is based on the calculated variations as coefficient of correlation variation (r).

The geNorm program calculates genes stability measure (M value) for a putative virulence gene based on the geometric mean of all studied genes. It relies on the principle that the expression ratio of two ideal virulence genes is identical in all samples, regardless of experimental conditions or cell type. It assumes that two genes that show the best correlation in their expression levels are the most appropriate ones and an expression factor can be calculated from these genes. Gene with the lowest M value is considered the most stable in expression. In our study, raw Ct values were transformed to quantities using the comparative Ct method, where the highest relative quantities (lowest Ct) for each genes. These linear scale data were used as input data, according to geNorm requirements. NormFinder calculates a stability value from a set of virulence genes. As input, it uses the same transformed data as geNorm. NormFinder calculates a stability value from eight virulence genes and it organizes them into different subgroups. This approach combines the intra-group and intergroup expression variation to a stability index that enables the ranking of genes by expression stability. A low numerical value for the stability index corresponds to a high stability in gene expression^17–20,33,34^.
